# Three new species of the planthopper genus *Sinonissus* Wang, Shi & Bourgoin, 2018 from southwest China (Hemiptera, Fulgoromorpha, Issidae)

**DOI:** 10.3897/zookeys.870.34417

**Published:** 2019-08-07

**Authors:** Zhi-Min Chang, Lin Yang, Jian-Kun Long, Xiang-Sheng Chen

**Affiliations:** 1 College of Animal Science, Guizhou University, Guiyang, Guizhou, 550025, China Guizhou University Guiyang China; 2 Institute of Entomology/Special Key Laboratory for Developing and Utilizing of Insect Resources, Guizhou University, Guiyang, Guizhou, 550025, China Guizhou University Guiyang China; 3 The Provincial Key Laboratory for Agricultural Pest Management of Mountainous Regions, Guizhou University, Guiyang, Guizhou, 550025, China Guizhou University Guiyang China

**Keywords:** Female genitalia, issid, Issini, morphology, Oriental region, taxonomy

## Abstract

The diagnostic characters of the Chinese planthopper genus *Sinonissus* Wang, Shi & Bourgoin, 2018 are redefined. Three new species of this genus, *S. daozhenensis* Chang & Chen, **sp. nov.** (Guizhou), *S. hamulatus* Chang & Chen, **sp. nov.** (Guizhou) and *S. longicaudus* Chang & Chen, **sp. nov.** (Sichuan) are described and illustrated, and their female genitalia compared. A checklist and key to the Chinese species of *Sinonissus* are given.

## Introduction

The family Issidae Spinola, 1839 is one of the largest planthopper families (Hemiptera, Fulgoromorpha), distributed in worldwide. Wang et al. (2016) proposed that the family Issidae was divided into Issinae Spinola, 1839 (including two tribes), Thioniinae Melichar, 1906 (including one tribe), and Hemisphaerinae, Melichar, 1906 (including four tribes), based on molecular data of 18S, 28S, COXI, and Cytb. The tribe Issini Spinola, 1839 was placed in the subfamily Issinae with Hysteropterini Melichar, 1906, but it differs from Hysteropterini by a number of characters: forewings with veins running in parallel; ScP+R, MP, and CuA bifurcated, the veins nearly reaching to the apical margin of the forewing; hind wings well developed or rudimentary; phallobase with one paired digitate processes on the inner side of the dorsolateral lobe (Gnezdilov 2003, 2016). Currently, the tribe Issini consists of four genera: *Issus* Brullé, 1832, *Latissus* Dlabola, 1974, *Issites* Haupt, 1956, and *Sinonissus* Wang, Shi & Bourgoin, 2018 (Gnezdilov and Bourgoin 2016; Wang et al. 2016; Wang et al. 2018). Wang et al. (2018) established the genus *Sinonissus* with one species from China (Sichuan, Chongqing).

The aim of this paper is to describe three new species of *Sinonissus* from China bringing the total number of species to four. Generic characteristics are redefined and a checklist and key to Chinese species of *Sinonissus* is provided.

## Materials and methods

The morphological terminology of the head and body follows Chan and Yang (1994) and Bourgoin et al. (2015), and the terminology of male and female genitalia follows Bourgoin (1993) and Gnezdilov (2002, 2003). Dry specimens were used for descriptions and illustrations. External morphology was observed under a stereoscopic microscope. All measurements are in millimeters (mm). The body measurements are from the apex of vertex to the tip of the forewings. The genital segments of the examined specimens were macerated in 10% NaOH, washed in water, and transferred to glycerin. Illustrations of the specimens were made with a Leica M125 and Olympus CX41 stereomicroscope. Photographs were taken with a Keyence VHX-1000C.

The type specimens and other examined specimens of the newly described species are all deposited in the Institute of Entomology, Guizhou University, Guiyang, China (**IEGU**).

## Taxonomy

### Genus Sinonissus

Taxon classificationAnimaliaIsopodaStyloniscidae

Wang, Shi & Bourgoin, 2018

EBE70264B0D75F3D81294AAC93E1FE08

Sinonissus Wang, Shi & Bourgoin, 2018: 53, figs 1–18.

#### Type species.

*Sinonissus brunetus* Wang, Shi & Bourgoin, 2018.

#### Diagnostic characters.

**Figures 1–8.**
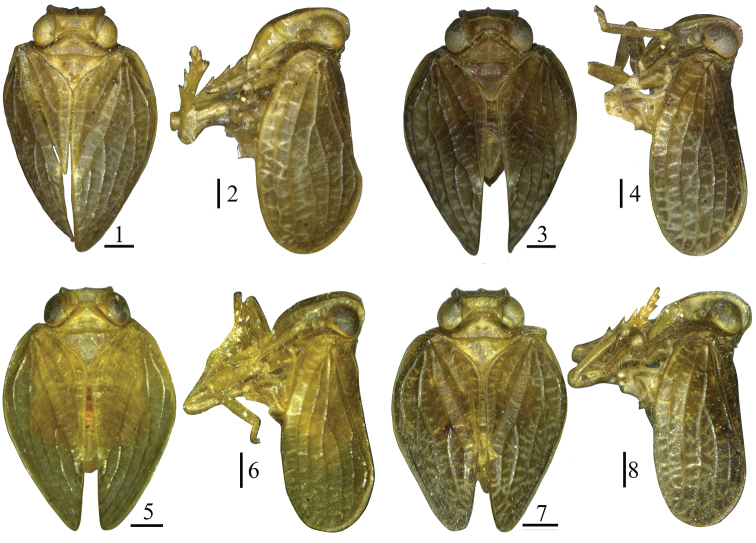
Habitus of *Sinonissus* species. **1, 2***Sinonissus brunetus* Wang, Shi & Bourgoin, 2018 **3, 4***Sinonissus daozhenensis* Chang & Chen, sp. nov. **5, 6***Sinonissus hamulatus* Chang & Chen, sp. nov. **7, 8***Sinonissus longicaudus* Chang & Chen, sp. nov. Scale bars: 0.5 mm.

**Figures 9–12.**
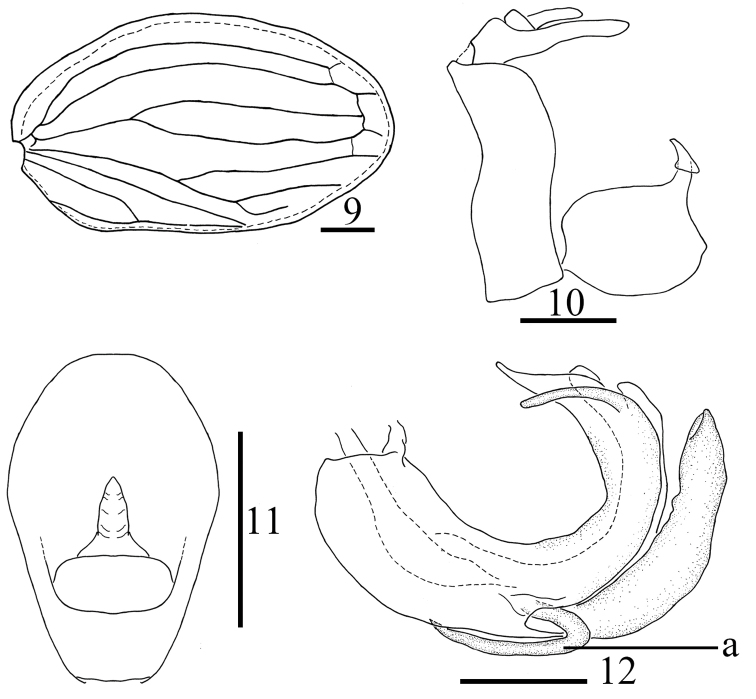
*Sinonissus brunetus* Wang, Shi & Bourgoin, 2018 **9** forewing **10** male genitalia, lateral view **11** anal segment, dorsal view **12** phallobase and aedeagus, lateral view. Scale bars: 0.5 mm. Abbreviations: a, short hooked process.

**Figures 13–22.**
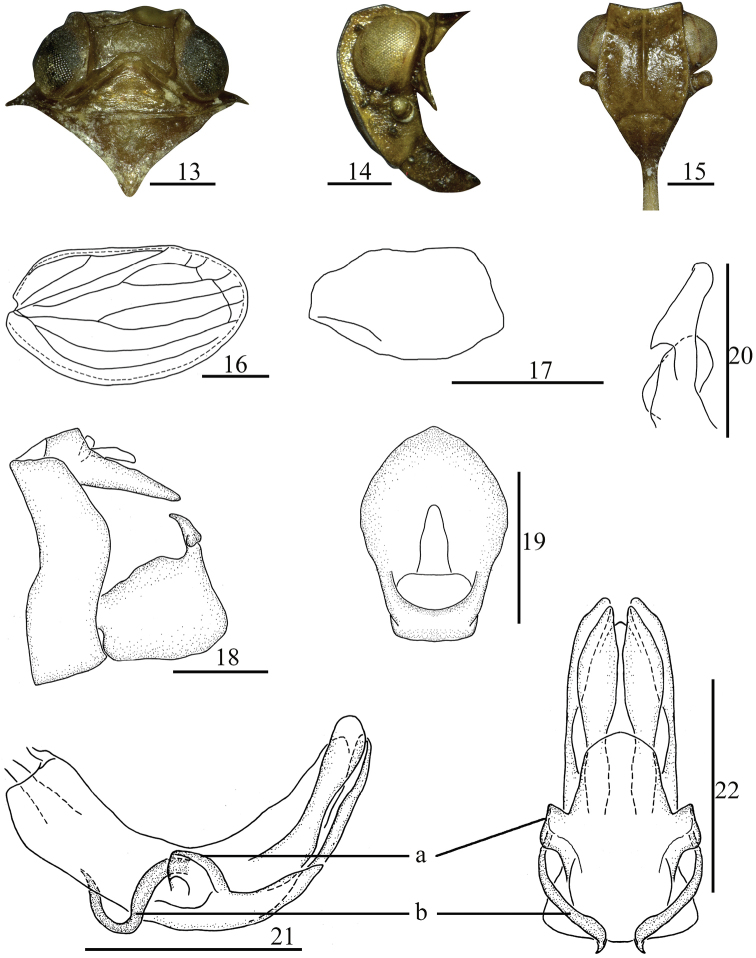
*Sinonissus daozhenensis* Chang & Chen, sp. nov. **13** head and thorax, dorsal view **14** head and thorax, lateral view **15** head, ventral view **16** forewing **17** hindwing **18** male genitalia, lateral view **19** anal segment, dorsal view **20** capitulum of genital styles, ventral view **21** phallobase and aedeagus, lateral view **22** phallobase and aedeagus, ventral view. Scale bars: 0.5 mm. Abbreviations: a, lobe-like process; b, long flexuous process.

Body small, slightly flat in vertical view. Width of head (Figs 1, 3, 5, 7) including eyes narrower than pronotum. Vertex (Figs 13, 32, 51) with width at base longer than length in middle ca. three times, disc of vertex depressed distinct, without median carina, anterior margin slightly convex or nearly straight, posterior margin obviously arched concave. Gena (Figs 14, 33, 52) with one obvious ocellus between compound eye and antenna in lateral view. Frons (Figs 15, 34, 53) rectangular, with median carina explicit, without lateral carina, the apical margin straight or not obviously forked, nearly reaching to frontoclypeal suture, with weeny tubercles near lateral margin, lateral margin nearly paralleled, the base slightly narrow, broader toward to apical margin, the widest below level of compound eyes. Clypeus (Figs 15, 34, 53) triangular, with median carina distinct or obscure. Rostrum surpassing mesotrochanters. Pronotum (Figs 13, 32, 51) without median carina or degraded, with lateral carina, without sub-lateral carina, pit each other between median carina and lateral carina, apical margin obtuse-angle concaved, posterior margin straight. Mesonotum (Figs 13, 32, 51) triangular, with median carina obvious or obscure or not, without sub-lateral carina. Forewings (Figs 9, 16, 35, 54) ovate, with length ca. 1.8 times longer than maximum width, anterior margin slightly cambered, anterior margin and posterior margin subparallel, apical margin obtusely rounded, longitudinal veins obvious and elevate, short transverse veins pale, with wide “hypocostal plate”, ScP and RP convergent near base, ScP vein long, no forked, nearly reaching the apical margin forewing, MP bifurcating two branches in basal 1/3, CuA forked into two branches near middle, behind the joint of Pcu and A_1_; CuP present, Pcu and A_1_ uniting in middle of clavus. Hindwings (Figs 17, 55) absent or reduced, small, vein simple. Hind tibiae each with two lateral spines, spinal formula of hind leg (7-9)–(8-9)–2.

#### Male genitalia.

Anal tube (Figs 11, 19, 37, 57) moderately long, irregularly pentagonal in dorsal view, the basal part narrow, the apical part more broad, maximum width in apical 1/3 of anal tube. Anal style (Figs 11, 19, 37, 57) moderately long, not surpassing anal tube. Pygofer (Figs 10, 18, 36, 56) symmetrical, irregularly rectangular; anterior margin and posterior margin nearly paralleled in lateral view, dorsal margin and ventral margin nearly paralleled in lateral view. Genital styles (Figs 10, 18, 38, 56) relatively rectangular, dorsal margin and ventral margin slightly arched, without triangular prominence near dorsal margin before capitulum. Capitulum of genital styles irregularly triangular, the basal part with half-elliptical process, the apical part with thin triangular process, neck obvious. Phallobase (Figs 12, 21, 40, 59) symmetrical, “U”-shaped tube in lateral view, dorsal lobe with apical part membranous, splitting into one sclerous branch in apical 1/3, with lateral lobe splitting into two branches, with ventral lobe shorter than dorsal lobe. Aedeagus (Figs 12, 21, 40, 59) with various long processes in lateral view.

#### Female genitalia

**Figures 23–31.**
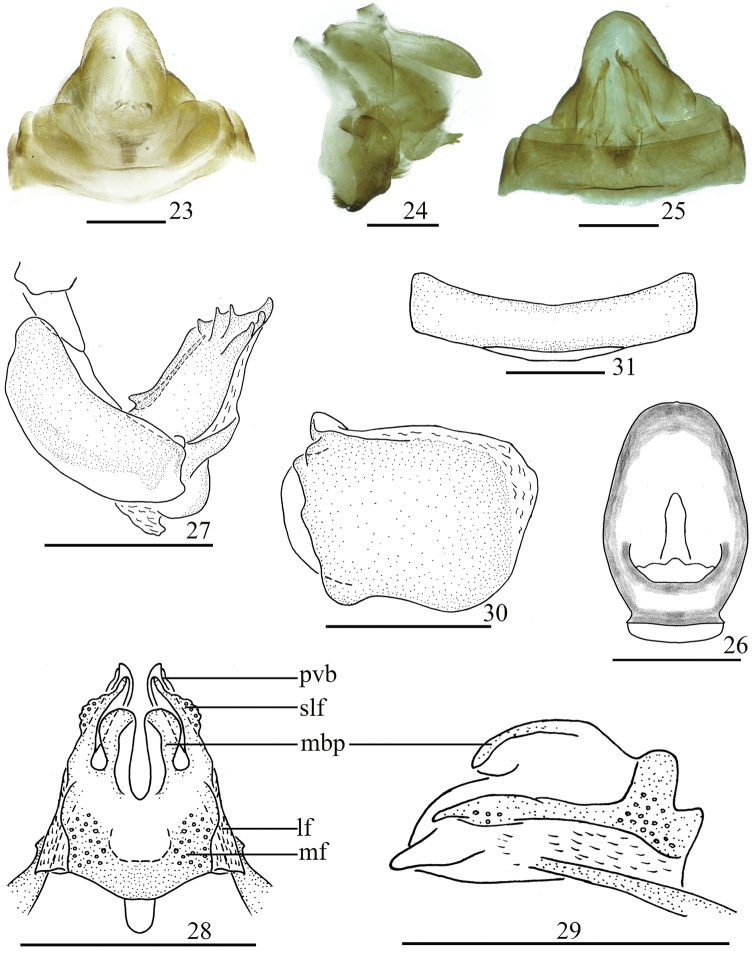
Female genitalia. *Sinonissus daozhenensis* Chang & Chen, sp. nov. **23** dorsal view **24** lateral view **25** ventral view **26** anal segment, dorsal view **27** anterior connective lamina of gonapophysis VIII, lateral view **28** posterior connective lamina of gonapophysis IX, dorsal view **29** posterior connective lamina of gonapophysis IX, lateral view **30** gonoplacs, lateral view **31** sternum VII, ventral view. Scale bars: 0.5 mm. Abbreviations: lf, lateral field of posterior connective lamina of gonapophysis IX; mdp, medial dorsal process; mf, medial field of posterior connective lamina of gonapophysis IX; pvd, posterior ventral lobes; slf, sublateral field of posterior connective lamina of gonapophysis IX.

(Figs 23–25, 42–44, 61–63). Anal tube (Figs 26, 45, 64) ovate, long in middle than the width, apical margin with unobvious or obvious membranous triangular protuberance. Anal style (Figs 26, 45, 64) long, located near base of anal tube. Hind margin of gonocoxa VIII with endogonocoxal lobe not obvious (Figs 27, 46, 65), endogonocoxal process membranous, narrowing gradually. Anterior connective lamina of gonapophysis VIII irregularly rectangular, with sclerous triangular process in basal dorsal margin, with two or three lateral teeth bearing two or three keels in lateral group and three teeth in apical group (Figs 27, 46, 65). Posterior connective lamina of gonapophysis IX (Figs 28, 29, 47, 48, 66, 67) triangular, with lateral field and sublateral field without obvious process (Figs 28, 47, 66); median field with prominence (median dorsal process) (Figs 28, 47, 66); ventroposterior lobes bent angle obtuse or acute (posterior ventral lobes) (Figs 29, 48, 67). Gonoplacs (Figs 30, 49, 68) without keels. Hind margin of sternum VII (Figs 31, 50, 69) median sunken, without any process in ventral view.

### Checklist of Chinese species of *Sinonissus* Wang, Shi & Bourgoin, 2018

*Sinonissus brunetus* Wang, Shi & Bourgoin, 2018; Chongqing, Guizhou, Sichuan.

*Sinonissus daozhenensis* Chang & Chen, sp. nov.; Guizhou.

*Sinonissus hamulatus* Chang & Chen, sp. nov.; Guizhou.

*Sinonissus longicaudus* Chang & Chen, sp. nov.; Sichuan.

#### Key to species of *Sinonissus* (based on males)

**Table:** 

1	Vertex with the width ca. 2.5 times as long as the middle line (Fig. 13)	***S. daozhenensis* sp. nov.**
–	Vertex with the width ca. 3.0 times as long as the middle line (Figs 32, 51)	**2**
2	Aedeagus with flagellate process in the middle in lateral view (Fig. 59); phallobase with ventral lobe triangular in ventral view (Fig. 60)	***S. longicaudus* sp. nov.**
–	Aedeagus with short hooked process in the middle in lateral view; phallobase with ventral lobe sub-rectangular in ventral view	**3**
3	Anal tube with spoon-like protrusion in lateral view (Fig. 36); aedeagus with short hooked process, tip of process directed to dorso-posterior (Fig. 40)	***S. hamulatus* sp. nov.**
–	Anal tube without spoon-like protrusion in lateral view (Figs 10, 11); aedeagus with short hooked process, tip of process directed to dorso-anterior (Fig. 12)	***S. brunetus***

#### Key to species of *Sinonissus* (based on females)

**Table:** 

1	Posterior connective lamina with median field with two ear-shaped prominences (Fig. 28)	***S. daozhenensis* sp. nov.**
–	Posterior connective lamina without the above characters	**2**
2	Posterior connective lamina with median field with near circular prominences (Fig. 47)	***S. hamulatus* sp. nov.**
–	Posterior connective lamina with median field with tubercle-like prominences (Fig. 66)	***S. longicaudus* sp. nov.**

**Notes**: The female of *S. brunetus* has not been examined, so this species cannot be included in the key.

### Sinonissus brunetus

Taxon classificationAnimaliaIsopodaStyloniscidae

Wang, Shi & Bourgoin, 2018

02452CCCA8DF5B0E95AD67CB16120EE1

Figs 1 , 2 , 9–12

Sinonissus brunetus Wang et al., 2018: 54: figs 1–18.

#### Material examined.

1♂, China: Guizhou, Chishui Alsophila National Nature Reserve (28°26'N, 106°01'E, 315 m), 28–29 May 2006, Y Tang leg..

#### Distribution.

China (Chongqing, Guizhou, Sichuan).

### Sinonissus daozhenensis

Taxon classificationAnimaliaIsopodaStyloniscidae

Chang & Chen sp. nov.

164B5D643EAA548AB200EF4F1F4DBE09

http://zoobank.org/1FF7BADC-7A3B-4665-B50F-91F090D2F252

Figs 3 , 4 , 13–22 , 23–31

#### Type material.

Holotype: ♂, China: Guizhou, Daozhen County, Sanqiao Town (29°03'N, 107°30'E, 1300–1600 m), 22–24 May 2004, B Zhang and P Xu leg.; paratypes: 3♀♀, same data as holotype; 1♂, Guizhou, Daozhen County, Sanqiao Town (29°03'N, 107°30'E, 1500 m), 23 May 2004, X-S Chen leg.; 2♀♀, Guizhou, Daozhen County, Dashahe National Nature Reserve (26°38'N, 108°03'E, 600–700 m), 25–27 May 2004, B Zhang and P Xu leg.; 1♀, Guizhou, Daozhen County, Sanqiao Town (29°03'N, 107°30'E, 600–900 m), 16 Sept. 2005, Q-Z Song leg..

#### Diagnosis.

This species is similar to *S. brunetus*, but it differs from the latter by phallobase with ventral lobe with lobe-like process near middle in lateral view (Fig. 21: a); aedeagus with long flexuous process near middle in lateral view (Fig. 21: b); posterior connective lamina of gonapophysis IX with median field with symmetrical ear-shaped prominences (Fig. 28).

#### Description.

Body length: male 3.85–4.04 mm, female 4.14–4.4 mm. Forewing: male 3.27–3.33 mm, female 3.38–3.54 mm.

***Coloration.*** General color brown (Figs 3, 4). Vertex, pronotum and mesonotum (Fig. 13) yellow brown. Eyes brown to black (Fig. 14). Forewings (Fig. 3) pale brown, longitudinal veins pale brown, transverse veins pale white. Hindwings brownish black. Legs yellow brown, with tips of spines on hind tibiae and tarsi black.

***Head and thorax.*** Head (Fig. 13) including eyes slightly narrower than pronotum (0.80: 1.00). Vertex (Fig. 13) shorter in middle than the wide at base (1.00: 2.52). Frons (Fig. 15) longer in midline than the widest breath (1.15: 1.00), median carina with the apical margin straight, nearly reaching to frontoclypeal suture. Clypeus (Fig. 15) triangular, with distinct median carina. Pronotum (Fig. 13) with median carina obscure, lateral carina reaching to the posterior margin. Mesonotum (Fig. 13) triangular, with median carina obscure. Forewings (Fig. 16) elongate, 1.67 times as long as maximum breadth. Hindwings (Fig. 17) reduced, small, with one vein. Hind tibiae each with two lateral spines, spinal formula of hind leg (8-9)–(8-9)–2.

***Male genitalia.*** Anal tube (Fig. 19) irregularly pentagonal in dorsal view, the widest in apical 1/3, longer in midline than the width (1.49: 1.00). Anal style (Fig. 19) sturdy and long, located at the base 1/3 of anal tube. Pygofer (Fig. 18) with dorsal margin slightly narrow than ventral margin, posterior margin convex in middle. Genital styles (Fig. 18) relatively rectangular, dorsal margin and ventral margin nearly parallel. Capitulum of genital styles relative long, irregularly triangular, neck obvious (Fig. 20). Phallobase (Figs 21, 22) with dorsal lobe cystiform at apical part, with stout rod-like process in apical half in lateral view; lateral lobe splitting into two stout branches; ventral lobe with one lobe-like process near middle in lateral view (Fig. 21: a), in ventral view, looking like three obvious lobes (Fig. 22: a), short, the apical margin arced convexly, reaching to 2/3 of dorsal lobe. Aedeagus with long flexuous process at middle in lateral view (Figs 21: b, 22: b).

***Female genitalia*** (Figs 23–31). Anal tube (Fig. 26) nearly oval, longer in middle than the widest breadth (1.61: 1.00), the apical margin arced, with unobvious membranous triangular protuberance, the widest at the basal 1/2. Anal style long, located at the basal 1/4 of anal tube (Fig. 26). Anterior connective lamina of gonapophysis VIII with obviously sclerous triangular process in basal dorsal margin, with three lateral teeth bearing three keels in lateral group and three apical teeth (Fig. 27). Posterior connective lamina of gonapophysis IX (Figs 28, 29) relative broad, median field symmetrical, with two ear-shaped prominences (medial dorsal process) (Fig. 28); ventroposterior lobes bent at obtuse angle (posterior ventral lobes) (Fig. 29). Gonoplacs (Fig. 30) without keels. Hind margin of sternum VII median slightly concaved in ventral view (Fig. 31).

**Figures 32–41.**
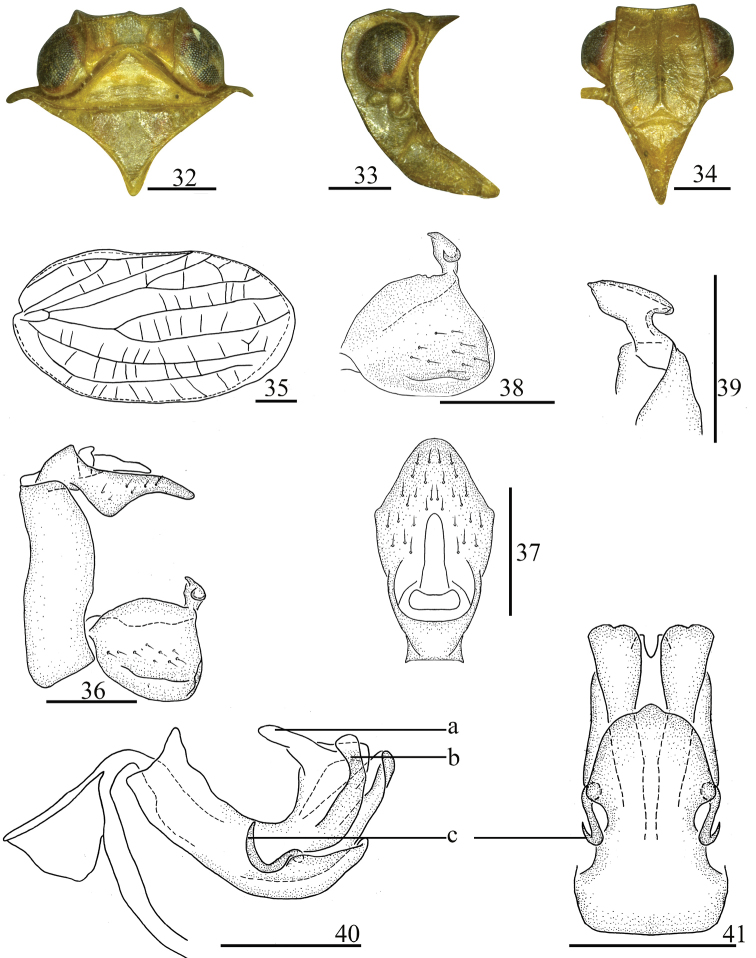
*Sinonissus hamulatus* Chang & Chen, sp. nov. **32** head and thorax, dorsal view **33** head and thorax, lateral view **34** head, ventral view **35** forewing **35** male genitalia, lateral view **37** anal segment, dorsal view **38** genital styles, lateral view **39** capitulum of genital styles, ventral view **40** phallobase and aedeagus, lateral view **41** phallobase and aedeagus, ventral view. Scale bars: 0.5 mm. Abbreviations: a, finger-like cystiform process; b, curved rod-like process; c, short hooked process.

#### Etymology.

The new species is named for its collecting location in the Daozhen County (Guizhou Province).

#### Host plant.

Bamboo (*Qiongzhuea communis* Hsueh & Yi).

#### Distribution.

China (Guizhou).

#### Remark.

This species is similar to *S. brunetus*, but it differs from the latter by: 1) the width of vertex (Fig. 13) as long as 2.52 times in middle line; 2) phallobase with ventral lobe with lobe-like process near middle in lateral view, in ventral view ventral lobe tree lobes (Figs 21, 22); 3) aedeagus with long flexuous process near middle in lateral view (Fig. 21); 4) female genitalia with posterior connective lamina of gonapophysis IX with median field with symmetrical ear-shaped prominences (Fig. 28).

### Sinonissus hamulatus

Taxon classificationAnimaliaIsopodaStyloniscidae

Chang & Chen sp. nov.

AA523653ACE55336BB95675EC2750881

http://zoobank.org/57E722C1-CB99-42BD-B7A9-2BC8FA0084E9

Figs 5 , 6 , 32–41 , 42–50

#### Type material.

Holotype: ♂, China: Guizhou, Jiangkou County, Fanjingshan National Nature Reserve (27°54'N, 108°38'E, 500–1800 m), 1–3 June 2002, X-S Chen leg.; paratypes: 10♂♂ 10♀♀, same data as holotype.

#### Diagnosis.

This species is similar to *S. brunetus*, but it differs from the latter by anal tube with spoon-like protrusion in lateral view (Fig. 36) (without spoon-like protrusion in *S. brunetus*); dorsal lobe of phallobase with rod-like cystiform processes at apical part (Fig. 40) (without rod-like process in *S. brunetus*); aedeagus with short hooked process, tip of process directed to dorso-posterior (Fig. 40) (tip of process directed to dorso-anterior in *S. brunetus*).

**Figures 42–50.**
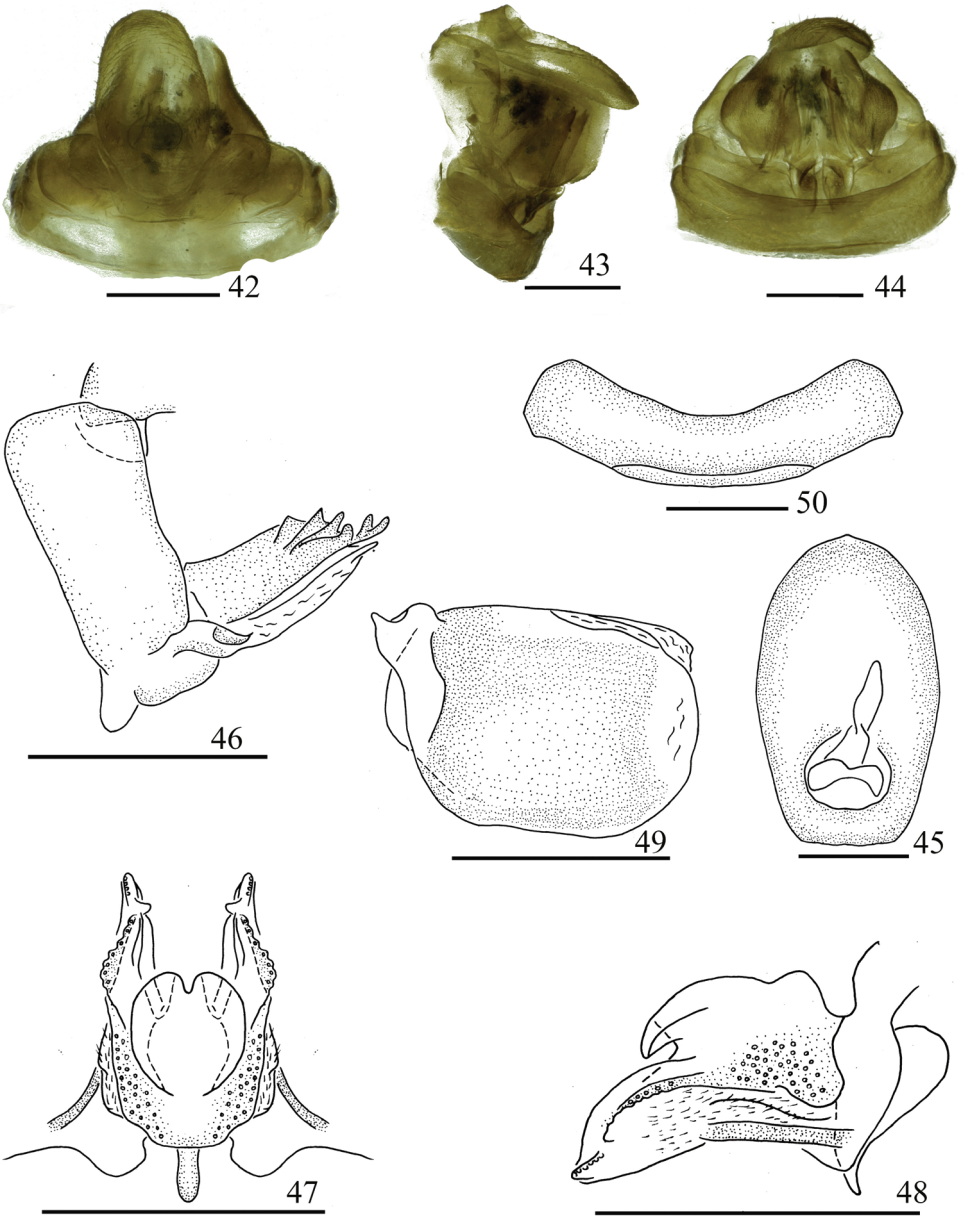
Female genitalia. *Sinonissus hamulatus* Chang & Chen, sp. nov. **42** dorsal view **43** lateral view **44** ventral view **45** anal segment, dorsal view **46** anterior connective lamina of gonapophysis VIII, lateral view **47** posterior connective lamina of gonapophysis IX, dorsal view **48** posterior connective lamina of gonapophysis IX, lateral view **49** gonoplacs, lateral view **50** sternum VII, ventral view. Scale bars: 0.5 mm.

**Figures 51–60.**
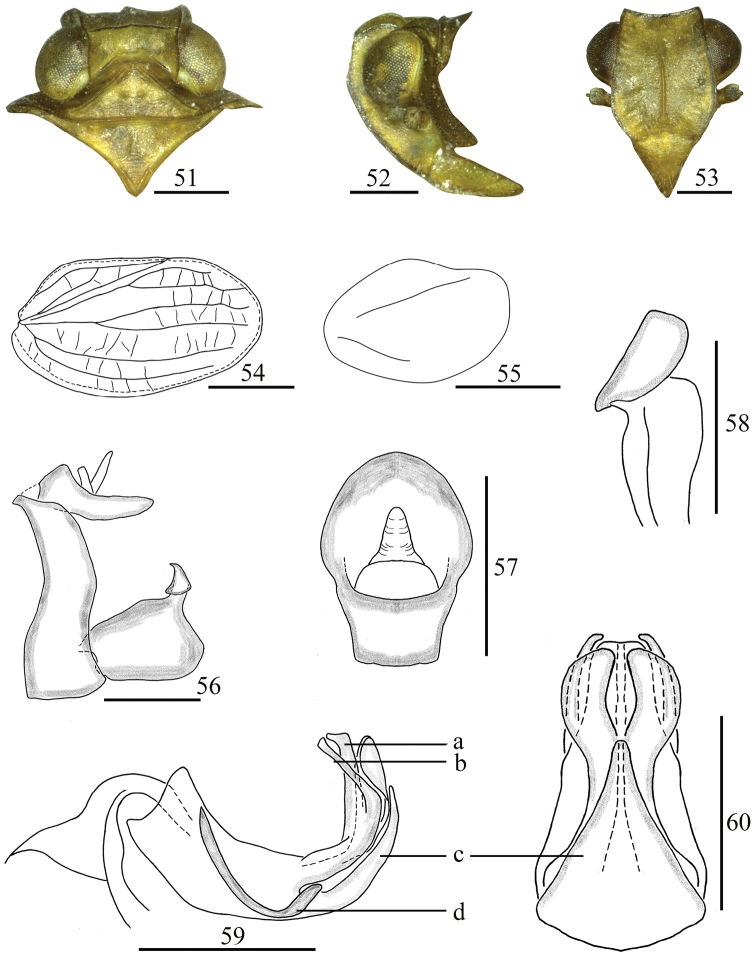
*Sinonissus longicaudus* Chang & Chen, sp. nov. **51** head and thorax, dorsal view **52** head and thorax, lateral view **53** head, ventral view **54** forewing **55** hindwing **56** male genitalia, lateral view **57** anal segment, dorsal view **58** capitulum of genital styles, ventral view **59** phallobase and aedeagus, lateral view **60** phallobase and aedeagus, ventral view. Scale bars: 0.5 mm. Abbreviations: a, horned cystiform process; b, thin rod-like process; c, ventral lobe process; d, long flagellate process.

#### Description.

Body length: male 3.61–3.98 mm, female 4.12–4.52 mm. Forewing: male 2.97–3.39 mm, female 3.46–3.80 mm.

***Coloration.*** General color pale yellow to yellowish brown (Figs 5, 6). Vertex, pronotum and mesonotum (Fig. 32) pale yellow. Eyes brown to black (Fig. 33). Forewings (Fig. 5) pale yellow or yellowish brown, longitudinal veins pale brown, transverse veins pale white. Legs yellow brown, with tips of spines on hind tibiae and tarsi black.

***Head and thorax.*** Head (Fig. 32) including eyes slightly narrower than pronotum (0.75: 1.00). Vertex (Fig. 32) shorter in middle than the wide at base (1.00: 3.00). Frons (Fig. 34) longer in middle than the widest breath (1.09: 1.00), median carina with the apical margin obscurely forked, nearly reaching to frontoclypeal suture. Clypeus triangular, with distinct median carina (Fig. 34). Pronotum (Fig. 32) with median carina obscure, lateral carina reaching to the posterior margin. Mesonotum (Fig. 32) triangular, without median carina. Forewings (Fig. 35) elongate, 1.82 times as long as maximum breadth. Hindwings absolutely reduced. Hind tibiae each with two lateral spines, spinal formula of hind leg 7–9–2.

***Male genitalia.*** Anal tube (Fig. 37) irregularly ligulate in dorsal view, the widest in apical 1/3, longer in midline than the width (1.85: 1.00), in lateral view anal tube with spoon-like protrusion (Fig. 36). Anal style (Fig. 37) sturdy and long, located at the base third of anal tube. Pygofer (Fig. 36) with dorsal margin slightly narrow than ventral margin. Genital styles (Fig. 38) relatively rectangular, dorsal margin slightly arched. Capitulum of genital styles relative long, irregularly triangular, neck obvious (Fig. 39). Phallobase (Figs 40, 41) with dorsal lobe finger-like cystiform process near apical part (Fig. 40: a), with relatively curved rod-like process in apical 1/3 (Fig. 40: b) in lateral view; lateral lobe splitting into two stout branches, the apical margin truncated in ventral view; ventral lobe short, unobvious lobe-like process near middle, reaching to 3/4 of dorsal lobe in lateral view, with three unobvious small lobes in ventral view. Aedeagus with short hooked process in the middle in lateral view, directed to dorso-posterior (Figs 40: c, 41: c).

***Female genitalia*** (Figs 42–50). Anal tube (Fig. 45) nearly oval, longer in middle than the widest breadth (1.70: 1.00), the apical margin arched, with unobvious membranous triangular protuberance, the widest at the basal 1/2. Anal style long, located at the basal fifth of anal tube (Fig. 45). Anterior connective lamina of gonapophysis VIII with obviously sclerous triangular process in basal dorsal margin, with three lateral teeth bearing three keels in lateral group and three apical teeth (Fig. 46). Posterior connective lamina of gonapophysis IX (Figs 47, 48) relatively broad, median field symmetrical, with nearly circular prominences, apical margin deeply incised in middle (medial dorsal process) (Fig. 47); ventroposterior lobes bent at obtuse angle (posterior ventral lobes), with membranous triangular process at inner region near the apical part (Fig. 48). Gonoplacs (Fig. 49) without keels. Hind margin of sternum VII median distinctly concave in ventral view (Fig. 50).

#### Etymology.

This new species is derived from the Latin word *hamulatus*, referring to the short hamular process of aedeagus.

#### Host plant.

Unknown.

#### Distribution.

China (Guizhou).

#### Remarks.

This new species is distinguished from other species of this genus by: 1) anal tube irregularly ligulate in dorsal view, with spoon-like protrusion in lateral view (Fig. 37); 2) phallobase with dorsal lobe rod-like cystiform processes at apical part, ventral lobes with three not obvious small lobes in ventral view (Fig. 40); 3) aedeagus with short hooked process in middle in lateral view (Fig. 40); 4) female genitalia with posterior connective lamina of gonapophysis IX in median field with nearly circular process (Fig. 47).

### Sinonissus longicaudus

Taxon classificationAnimaliaIsopodaStyloniscidae

Chang & Chen sp. nov.

1F35D66B216E55059261C358A3A07548

http://zoobank.org/730FE54F-DB72-4DC8-9A13-69C16ED1A8DD

Figs 7 , 8 , 51–60 , 61–69

#### Type material.

Holotype: ♂, China: Sichuan, Emeishan, Da’e Village (29°33'N, 103°24'E), 12–14 July 2010, Y-L Zheng leg.; paratypes: 1♂6♀♀, same data as holotype, Y-L Zheng and P Zhang leg.; 1♂1♀, Sichuan, Emeishan (29°30'N, 103°20'E), 3 Aug. 2012, H Li leg..

#### Diagnosis.

This species is similar to *S. brunetus* but can be distinguished from the latter by aedeagus (Fig. 59) with long flagellate process in the middle in lateral view; phallobase (Fig. 60) with ventral lobe triangular, apical margin extremely narrow, the basal part broad in ventral view.

#### Description.

Body length: male 3.38–3.63 mm, female 4.05–4.36 mm. Forewing: male 2.74–3.01 mm, female 3.27–3.62 mm.

***Coloration.*** General color pale yellow to pale yellowish brown (Figs 7, 8). Vertex, pronotum and mesonotum (Fig. 51) pale yellow. Eyes brown (Fig. 52). Forewings (Fig. 7) pale yellow or yellowish brown, longitudinal veins pale brown, transverse veins pale white. Legs yellow brown, with tips of spines on hind tibiae and tarsi black.

***Head and thorax.*** Head (Fig. 51) including eyes slightly narrower than pronotum (0.73: 1.00). Vertex (Fig. 51) shorter in middle than the wide at base (1.00: 3.33). Frons (Fig. 53) longer in middle than the widest breath (1.14: 1.00), median carina with the apical margin straight, nearly reaching to frontoclypeal suture. Clypeus triangular, with obscure median carina (Fig. 53). Pronotum (Fig. 51) with median carina obscure, lateral carina reaching to the posterior margin. Mesonotum (Fig. 51) triangular, with median carina obvious. Forewings (Fig. 54) elongate, 1.78 times as long as maximum breadth. Hindwings (Fig. 55) reduced, small, with two veins. Hind tibiae each with two lateral spines, spinal formula of hind leg 8–8–2.

***Male genitalia.*** Anal tube (Fig. 57) irregularly pentagonal in dorsal view, widest in the middle, longer in midline than the width (1.41: 1.00), ventral margin nearly straight. Anal style sturdy and short, located at the base half of anal tube (Fig. 57). Pygofer (Fig. 56) with dorsal margin narrower than ventral margin. Genital styles (Fig. 56) rectangular, dorsal margin and ventral margin nearly parallel. Capitulum of genital styles relatively short, irregularly triangular, neck obvious (Fig. 58). Phallobase (Figs 59, 60) with dorsal lobe small horned cystiform process at apical part (Fig. 59: a), with relatively straight thin rod-like process in apical 1/4 (Fig. 59: b) in lateral view; lateral lobe splitting into two stout branches, the apical margin arced in ventral view; ventral lobe short, reaching to 5/6 of dorsal lobe in lateral view, in ventral view the apical part triangular, apical margin extremely narrow, the basal part broad (Figs 59: c, 60: c). Aedeagus with long flagellate process in the middle in lateral view, directed to dorso-anterior (Fig. 59: d).

**Figures 61–69.**
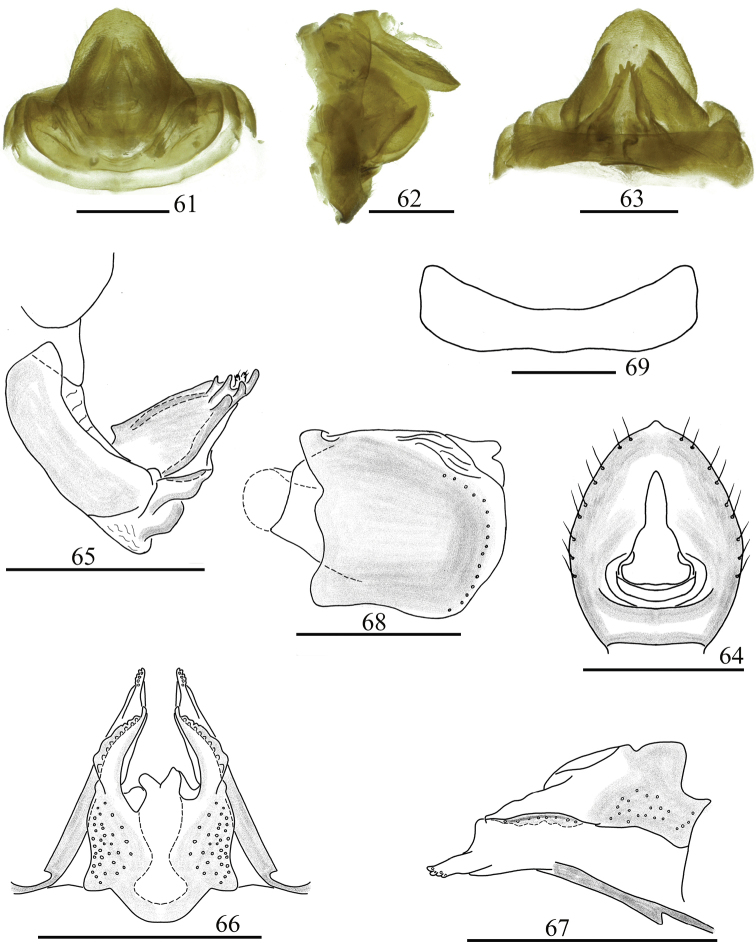
Female genitalia. *Sinonissus longicaudus* Chang & Chen, sp. nov. **61** dorsal view **62** lateral view **63** ventral view **64** anal segment, dorsal view **65** anterior connective lamina of gonapophysis VIII, lateral view **66** Posterior connective lamina of gonapophysis IX, dorsal view **67** Posterior connective lamina of gonapophysis IX, lateral view **68** Gonoplacs, lateral view **69** Sternum VII, ventral view. Scale bars: 0.5 mm.

***Female genitalia*** (Figs 61–69). Anal tube (Fig. 64) nearly oval, longer in middle than the widest breadth (1.29: 1.00), the apical margin arced, with obvious membranous triangular protuberance, the widest near the basal 1/3. Anal style long, located at the basal third of anal tube (Fig. 64). Anterior connective lamina of gonapophysis VIII with obviously sclerous triangular process in basal dorsal margin, with two lateral teeth bearing two or three keels in lateral group and two or three apical teeth (Fig. 65). Posterior connective lamina of gonapophysis IX (Figs 66, 67) relatively narrow, median field asymmetrical, with tubercle-like prominences (medial dorsal process) (Fig. 66); ventroposterior lobes bent at acute angle (posterior ventral lobes) (Fig. 67). Gonoplacs (Fig. 68) without keels. Hind margin of sternum VII median distinctly concave in ventral view (Fig. 69).

#### Etymology.

This new species is named for the presence of one long flagellate process of aedeagus.

#### Host plant.

Unknown.

#### Distribution.

China (Sichuan).

#### Remarks.

This new species is distinguished from other species of this genus by: 1) aedeagus with long flagellate process in the middle in lateral view, directed to dorso-anterior (Fig. 59: d); 2) phallobase with dorsolateral lobe relatively straight, thin, rod-like process in lateral view (Fig. 59: b), ventral lobes triangular, apical margin extremely narrow, basal part broad in ventral view (Fig. 60: c); 3) female genitalia with posterior connective lamina of gonapophysis IX median field asymmetrical, with tubercle-like prominences (Fig. 66).

## Discussion

**Figure 70.**
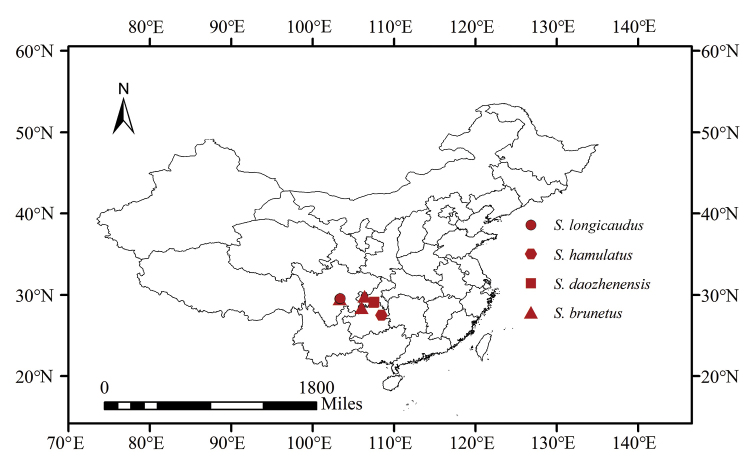
Geographical distribution of *Sinonissus*.

According to the geographic distribution of the four species of *Sinonissus*, all species are distributed in the Oriental region and appear to be found only in China (Fig. 70). Following the taxonomic system of Gnezdilov (2002, 2003, 2009, 2013), the family Issidae consists of three tribes: Issini Spinola, 1839, Hemisphaeriini Melichar, 1906, and Parahiraciini Cheng & Yang, 1991, all in the subfamily Issinae, and the genera *Sinonissus* and *Celyphoma* Emeljanov, 1971 may also be placed in Issini Spinola, 1839, due to the genera having the following characters in common: the small body size, the vertex with its width longer along the midline, the forewing with veins running in parallel, the hind wing reduced or absent, and the phallobase without paired digitate processes on the inner side of the dorsolateral lobe.

Wang et al. (2016, 2018) placed *Sinonissus* in the Issini and *Celyphoma* in Hysteropterini in a new taxonomic system, based on limited molecular data. According to Bourgoin (2018), the subfamily Issinae contains the tribe Issini (including 61 genera) and Hysteropterini (including four genera) in the world. The subfamily Issinae is characteristically distributed in Wallace’s Palaearctic region. In China, there are two genera recorded in the tribe Issini, *Issus* Brullé, 1832, and *Sinonissus* Wang, Shi & Bourgoin, 2018, and two genera in Hysteropterini: *Celyphoma* Emeljanov, 1971, *Potaninum* Gnezdilov, 2017. Fewer species were recorded in the subfamily Issinae: *Issus coleoptratus* (Fabricius, 1781) distributed in Hong Kong and four species of *Sinonissus* in Sichuan, Chongqing, and Guizhou (see Fig. 70). Gnezdilov (2017) recorded *Potaninum boreale* (Melichar, 1902) in Sichuan; four species of *Celyphoma* Emeljanov, 1971 have been recorded in Gansu, Inner Mongolia, Ningxia, Qinghai, and Xingjiang (Meng and Wang 2012, Chen et al. 2014). Except *Celyphoma*, other species and genera of Issinae in China are distributed in the Oriental region. Thus, the phylogeny based on the geographical distribution of Issinae is unstable and paradoxical.

**Figures 71–74.**
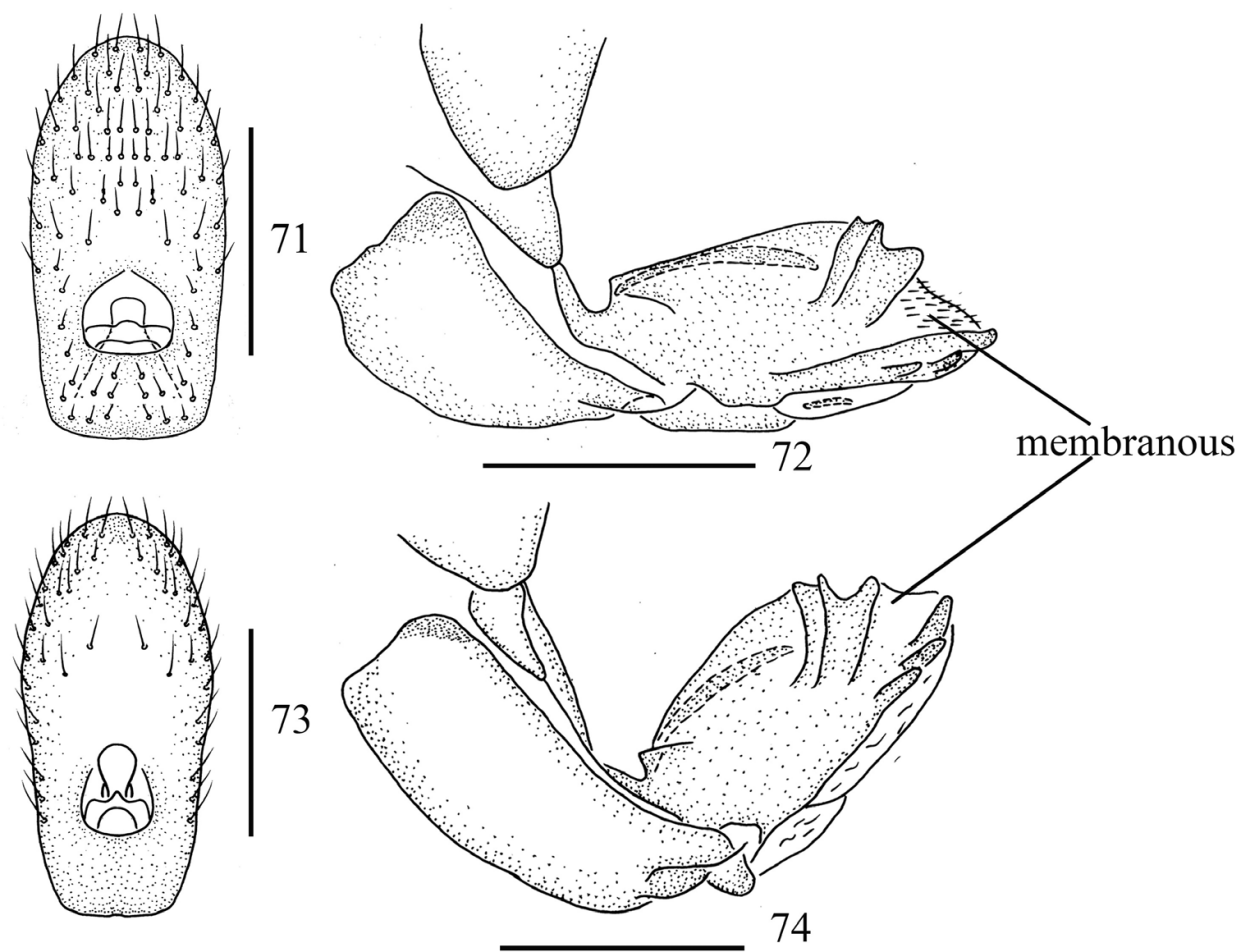
Female genitalia. **71, 72***Celyphoma gansua* Chen, Zhang & Chang, 2014 **73, 74***Celyphoma yangi* Chen, Zhang & Chang, 2014 **71, 73** anal segment, dorsal view **72, 74** anterior connective lamina of gonapophysis VIII, lateral view. Scale bars: 0.5 mm.

For the female genitalia of Issinae, only female genitalia of *Sinonissus* and *Celyphoma* were examined in this work. *Sinonissus* is obviously different from *Celyphoma* in the anal tube having an apical margin and an obvious membranous triangular protuberance, widest near the basal half; the anal style is long (Figs 26, 45, 64) [anal tube with apical margin without triangular protuberance, lateral margin parallel, anal style short in *Celyphoma* (Figs 71, 73)]; and the anterior connective lamina of gonapophysis VIII has the apical part sclerous (Figs 27, 46, 65) [membranous in *Celyphoma* (Figs 72, 74)].

The phylogeny based on a combination of the geographical distribution, male and female characters, and molecular data may be more convincing. Unfortunately, there is no further morphological information and molecular data is unavailable.

## Supplementary Material

XML Treatment for Sinonissus

XML Treatment for Sinonissus brunetus

XML Treatment for Sinonissus daozhenensis

XML Treatment for Sinonissus hamulatus

XML Treatment for Sinonissus longicaudus

## References

[B1] Bourgoin T (1993) Female genitalia in HemipteraFulgoromorpha, morphological and phylogenetic data. Annales de la Société Entomologique France 93: 225–244.

[B2] Bourgoin T (2018) FLOW (Fulgoromorpha lists on the Web): a world knowledge base dedicated to Fulgoromorpha Version 8, updated 8 May 2019. http://hemiptera-databases.org/flow/

[B3] Bourgoin T Wang RR Asche M Hoch H Soulier-Perkins A Stroiński A Yap S Szwedo J (2015) From micropterism to hyperpterism: recognition strategy and standardized homology-driven terminology of the forewing venation patterns in planthoppers (Hemiptera: Fulgoromorpha). Zoomorphology 134(1): 63–77. 10.1007/s00435-014-0243-6 25705075 PMC4326643

[B4] Chan ML Yang CT (1994) Issidae of Taiwan (Homoptera: Fulgoroidea). Chen Chung Book, Taichung, 188 pp.

[B5] Chen XS Zhang ZG Chang ZM (2014) Issidae and Caliscelidae (Hemiptera: Fulgoroidea) from China. Guizhou Science and Technology Publishing House, Guiyang, 242 pp.

[B6] Gnezdilov VM (2002) Morphology of the ovipositor in members of the subfamily Issinae (Homoptera, Cicadina, Issidae). Entomologicheskoe Obozrenie 81(3): 605–626.

[B7] Gnezdilov VM (2003) Review of the family Issidae (Homoptera, Cicadina) of the European fauna, with notes on the structure of ovipositor in planthoppers. Chteniya pamyati N.A. Kholodkovskogo (Meetings in memory of NA Cholodkovsky) 56(1): 1–145.

[B8] Gnezdilov VM (2009) Revisionary notes on some tropical Issidae and Nogodinidae (Hemiptera: Fulgoroidea). Acta Entomologica Musei Nationalis Pragae 49(1): 75–92.

[B9] Gnezdilov VM (2013) Revision of the tribe Colpopterini Gnezdilov, 2003 (Homoptera, Fulgoroidea: Nogodinidae). Entomological Review 93(3): 337–353. 10.1134/S0013873813030081

[B10] Gnezdilov VM (2016) Notes on the phylogenetic relationships of planthoppers of the family Issidae (Hemiptera, Fulgoroidea) of the Western Palaearctic fauna, with descriptions of two new genera. Entomologicheskoe Obozrenie 95(2): 362–382. 10.1134/S0013873816030106

[B11] Gnezdilov VM (2017) A new genus for *Hysteropterum boreale* Melichar, 1902 (Hemiptera, Auchenorrhyncha: Fulgoroidea: Issidae) from China, Entomological Review 97(1): 57–61. 10.1134/S0013873817010079

[B12] Gnezdilov VM Bourgoin T (2016) On the taxonomic position of *Issus reticulatus* Bervoets, 1910 (Hemiptera: Fulgoroidea: Issidae) from Baltic Amber. Entomological Review 96(5): 631–633. 10.1134/S0013873816050092

[B13] Meng R Wang YL (2012) Two new species of the genus *Celyphoma* Emeljanov, 1971 (Hemiptera: Fulgoromorpha: Issidae) from China. Zootaxa 3497: 17–28. 10.11646/zootaxa.3497.1.2

[B14] Wang ML Shi AM Bourgoin T (2018) Morphological and molecular data reveal a new genus of the tribe Issini from Southern China (Hemiptera, Fulgoromorpha, Issidae). ZooKeys 766: 51–61. 10.3897/zookeys.766.24299 PMC6010510 29930478

[B15] Wang ML Zhang YL Bourgoin T (2016) Planthopper family Issidae (Insecta: Hemiptera: Fulgoromorpha): linking molecular phylogeny with classification. Molecular Phylogenetics and Evolution 105: 224–234. 10.1016/j.ympev.2016.08.012 27554758

